# Mutation in MRPS34 Compromises Protein Synthesis and Causes Mitochondrial Dysfunction

**DOI:** 10.1371/journal.pgen.1005089

**Published:** 2015-03-27

**Authors:** Tara R. Richman, Judith A. Ermer, Stefan M. K. Davies, Kara L. Perks, Helena M. Viola, Anne-Marie J. Shearwood, Livia C. Hool, Oliver Rackham, Aleksandra Filipovska

**Affiliations:** 1 Harry Perkins Institute of Medical Research, Centre for Medical Research, QEII Medical Centre, The University of Western Australia, Nedlands, Western Australia, Australia; 2 School of Anatomy, Physiology and Human Biology, The University of Western Australia, Crawley, Western Australia, Australia; 3 Victor Chang Cardiac Research Institute, Darlinghurst, New South Wales, Australia; 4 School of Chemistry and Biochemistry, The University of Western Australia, Crawley, Western Australia, Australia; University of Miami, UNITED STATES

## Abstract

The evolutionary divergence of mitochondrial ribosomes from their bacterial and cytoplasmic ancestors has resulted in reduced RNA content and the acquisition of mitochondria-specific proteins. The mitochondrial ribosomal protein of the small subunit 34 (MRPS34) is a mitochondria-specific ribosomal protein found only in chordates, whose function we investigated in mice carrying a homozygous mutation in the nuclear gene encoding this protein. The *Mrps34* mutation causes a significant decrease of this protein, which we show is required for the stability of the 12S rRNA, the small ribosomal subunit and actively translating ribosomes. The synthesis of all 13 mitochondrially-encoded polypeptides is compromised in the mutant mice, resulting in reduced levels of mitochondrial proteins and complexes, which leads to decreased oxygen consumption and respiratory complex activity. The *Mrps34* mutation causes tissue-specific molecular changes that result in heterogeneous pathology involving alterations in fractional shortening of the heart and pronounced liver dysfunction that is exacerbated with age. The defects in mitochondrial protein synthesis in the mutant mice are caused by destabilization of the small ribosomal subunit that affects the stability of the mitochondrial ribosome with age.

## Author Summary

Mitochondria make most of the energy required by eukaryotic cells and therefore they are essential for their normal function and survival. Mitochondrial function is regulated by both the mitochondrial and nuclear genome. Mutations in nuclear genes encoding mitochondrial proteins lead to mitochondrial dysfunction and consequently diminished energy production, a major symptom of metabolic and mitochondrial diseases. The molecular mechanisms that regulate mitochondrial gene expression and how dysfunction of these processes causes the pathologies observed in these diseases are not well understood. Messenger RNAs encoded by mitochondrial genomes are translated on mitochondrial ribosomes that have unique structure and protein composition. Mitochondrial ribosomes are a patchwork of core proteins that share homology with prokaryotic ribosomal proteins and mitochondria-specific proteins, which can be unique to different organisms. Mitochondria-specific ribosomal proteins have key roles in disease however their functions within mitochondria are not known. Here we show that a point mutation in a mammalian-specific ribosomal protein causes mitochondrial dysfunction, heart abnormalities and progressive liver disease. This mouse provides a valuable model to elucidate the pathogenic mechanisms and progression of metabolic diseases with age, while enabling a more thorough understanding of mitochondrial ribosomes and protein synthesis.

## Introduction

Mitochondria are composed of proteins encoded by the nuclear and mitochondrial genomes. Most of the mitochondrial proteins including the ribosomal proteins and translation factors that are responsible for the expression of the mitochondrial genome are synthesized on cytoplasmic ribosomes and imported into mitochondria post-translationally. In chordates, the mitochondrial genome encodes 22 tRNAs, 2 rRNAs and 11 mRNAs that are translated on mitochondrial ribosomes (mitoribosomes) into 13 polypeptides, all members of the oxidative phosphorylation complexes [1]. Mutations in mitochondrial genes or nuclear genes coding for mitochondrial proteins result in mitochondrial dysfunction and impaired energy production that cause mitochondrial diseases (reviewed in [2]). The most common cause of mitochondrial diseases are defects in the translational machinery (reviewed in [3]), however the mechanisms of mitochondrial protein synthesis are not well understood.

Mammalian mitoribosomes are 55S particles consisting of a 28S small subunit that includes the 12S rRNA and ~ 29 proteins and a 39S large subunit, which contains the 16S rRNA and ~ 48 proteins [4–7]. Mitoribosomes are distinct from their bacterial and cytoplasmic counterparts; they have reduced RNA content and an increased number of proteins [8]. The mitoribosome consists of proteins that share homology with bacterial ribosomes, some of which can have mitochondria-specific extensions, and additional, mitochondria-specific proteins that decorate the ribosomal surface, the mRNA entrance site and form some of the bridges linking the small and large subunits [6,7,9,10]. The increased ribosomal protein content does not entirely compensate for the loss of rRNA as many of the mitochondria-specific ribosomal proteins do not replace the missing RNA helices but instead have unique positions on the exterior of the mitochondrial ribosome [11,12]. Recent cryo-electron microscopy (cryo-EM) reconstructions indicate that the additional protein elements may fulfil roles necessitated by the unique features of the mitochondrial leaderless mRNAs, likely in their recognition, as well as facilitating the translation of the particularly hydrophobic proteins they encode and making the contacts between the small and large ribosomal subunits [6,7,9,10,12]. The mitoribosomes are located inside the matrix, the site of the transcriptome, and associate closely with the mitochondrial inner membrane through MRPL45 [7]. This positioning allows for co-translational insertion of the hydrophobic proteins, which are translated by the mitoribosome, into the inner membrane and their assembly into oxidative phosphorylation (OXPHOS) complexes [13].

There is very little known about the functions and roles of the mitochondria-specific ribosomal proteins, therefore characterizing their function within the ribosome and mitochondria will provide valuable insights into mitochondrial translation. The mitochondrial ribosomal protein of the small subunit 34 (MRPS34) has been identified as one of 15 mitochondria-specific proteins that are part of the small ribosomal subunit [14,15]. Although MRPS34 has been found localized to mitochondria and associated with the human homolog of the *Drosophila* discs large tumor suppressor protein (hDLG) [16], its role in mitochondria and protein synthesis has not been identified or characterized. Here we investigated the role of MRPS34 in mice carrying a homozygous mutation in the nuclear gene encoding this protein that causes a significant decrease of this protein. MRPS34 is required for protein synthesis of all 13 mitochondrially-encoded polypeptides and stability of the 12S rRNA and specific mRNAs. Dysfunction in the efficiency of mitochondrial protein synthesis leads to reduced mitochondrial oxygen consumption and respiratory complex activity in the mutant mice indicating that MRPS34 is essential for the stability of actively translating ribosomes. The *Mrps34* mutation causes tissue-specific molecular and pathological changes that result in alterations in fractional shortening of the heart and pronounced liver steatosis that leads to fibrosis with age. Mitochondrial dysfunction caused by the *Mrps34* mutation is likely caused by decreased levels of mitochondrial ribosomal subunits and translationally competent mitoribosomes.

## Results

### *Mrps34* ^*mut/mut*^ mice have reduced expression of the MRPS34 protein

**Fig 1:**
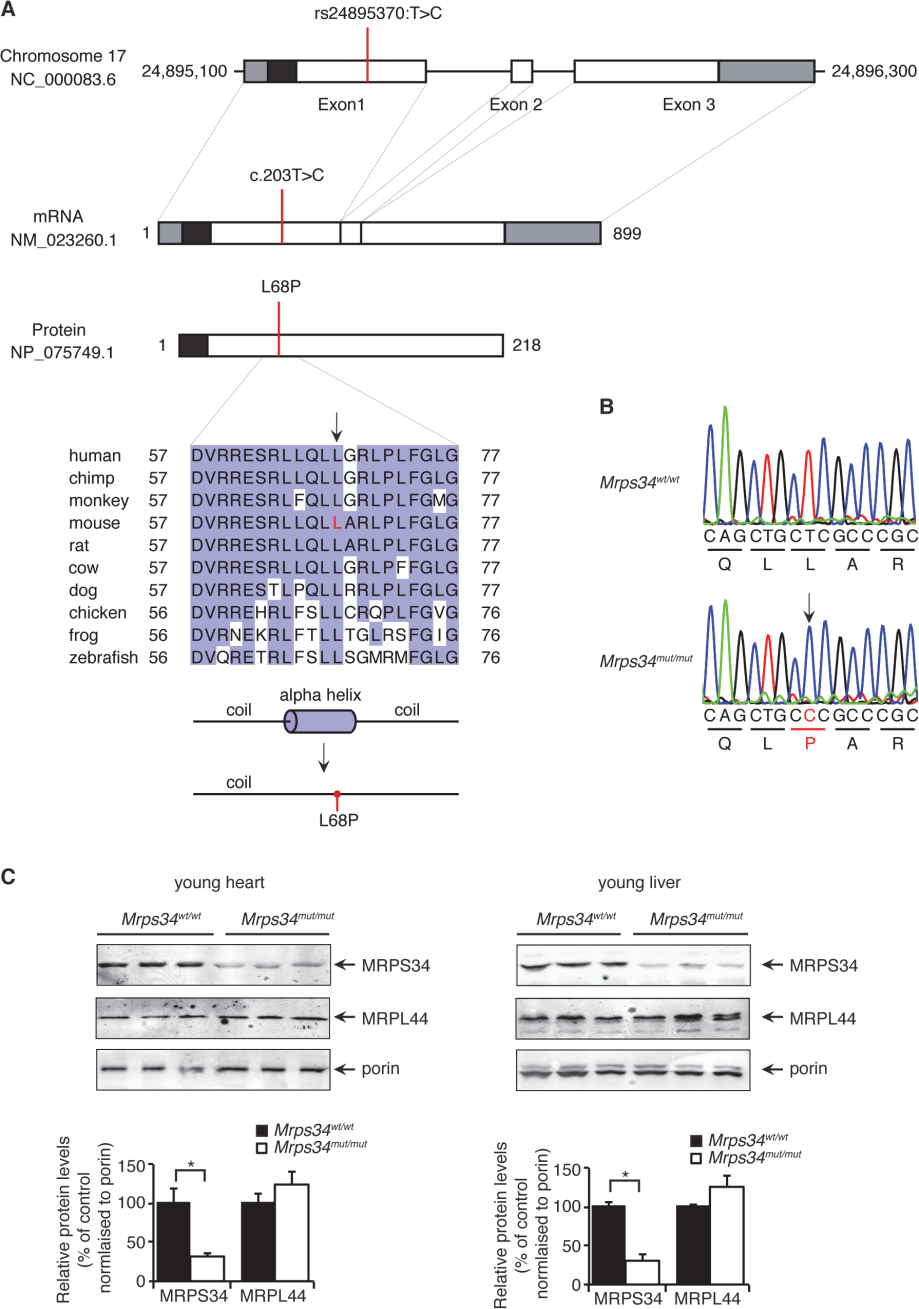
A homozygous point mutation in the *Mrps34* gene causes a decrease in the MRPS34 protein. (A) Schematic showing the location of the mutation in the *Mrps34* gene, mRNA and protein. The 5′- and 3′-untranslated regions are shown as grey boxes and the predicted mitochondrial targeting sequence is shown as a black box (predicted using MitoProtII, [40]). Conservation of the protein sequence surrounding the mutation is highlighted with residues identical to those in the mouse sequence boxed (the mutated leucine is shown in red in the mouse sequence). Sequences used were obtained from GenBank at NCBI (human, *Homo sapiens*, NP_076425.1; chimp, *Pan troglodytes*, XP_001160122.1; monkey, *Macaca mulatta*, NP_001244544.1; mouse, *Mus musculus*, NP_081622.1; rat, *Rattus norvegicus*, NP_001099241.1; cow, *Bos taurus*, NP_001192540.1; dog, *Canis lupus familiaris*, XP_003639160.1; chicken, *Gallus gallus*, NP_001264526.1; frog, *Xenopus tropicalis*, NP_001004910.2; zebrafish, *Danio rerio*, NP_001007377.2) and the alignment was produced using ClustalW2 [41]. The secondary structure of mouse MRPS34 was predicted using PSI-PRED [42] and the mutated region is shown. An alpha helical region is predicted to be lost when the L68P mutation is present. (B) The mutation was confirmed by Sanger sequencing of PCR amplicons from *Mrps34* ^*mut/mut*^ and littermate matched *Mrps34* ^*wt/wt*^ mice. (C) Mutation in the nuclear gene encoding the mitochondrial MRPS34 protein leads to its decreased abundance. MRPS34 and MRPL44 protein levels were investigated in mitochondria isolated from hearts and livers of *Mrps34* ^*wt/wt*^ and *Mrps34* ^*mut/mut*^ mice by immunoblotting. Porin was used as a loading control. The data are representative of results obtained from at least 8 mice from each strain. Data are means ± SEM of three separate experiments; *, *p* < 0.05 compared with control treatments by a 2-tailed paired Student’s *t* test.

A mouse line carrying an ENU-induced T203C point mutation in the *Mrps34* gene that converts a leucine residue at position 68 to proline (Fig. 1A) was identified by whole exome sequencing [17]. Sanger sequencing shows that the *Mrps34* ^*mut/mut*^ mice are homozygous for the mutation that is absent in age and littermate matched control *Mrps34* ^*wt/wt*^ mice (Fig. 1B). The leucine residue at position 68 is conserved in vertebrates (Fig. 1A), and mutation to proline is predicted to disrupt the formation of an alpha helix. To determine whether the mutation caused changes in the abundance of the MRPS34 protein we carried out immunoblotting of mitochondrial lysates isolated from liver and heart of *Mrps34* ^*mut/mut*^ and *Mrps34* ^*wt/wt*^ mice. The MRPS34 protein was reduced in the heart and liver of the mutant mice (Fig. 1C), indicating that the mutation causes instability of the protein. The MRPS34 protein is expressed in all examined tissues including brain, colon, heart, kidney, liver, thymus, pancreas, skin and testis and the *Mrps34* mutation results in decreased levels of the protein in these tissues (S1 Fig). Mutations in nuclear genes encoding proteins that are part of the translational machinery have been shown to cause mitochondrial diseases with varying age of onset and diverse clinical pathologies that affect a range of different tissues [2,3]. Next we sought to determine the effects of the *Mrps34* mutation, in young (6–8 week) and aged (28–30 week) mice, on mitochondrial function and the downstream effects on energy metabolism and disease pathology.

### The *Mrps34* mutation affects stability of the 12S rRNA and specific mitochondrial mRNAs

**Fig 2:**
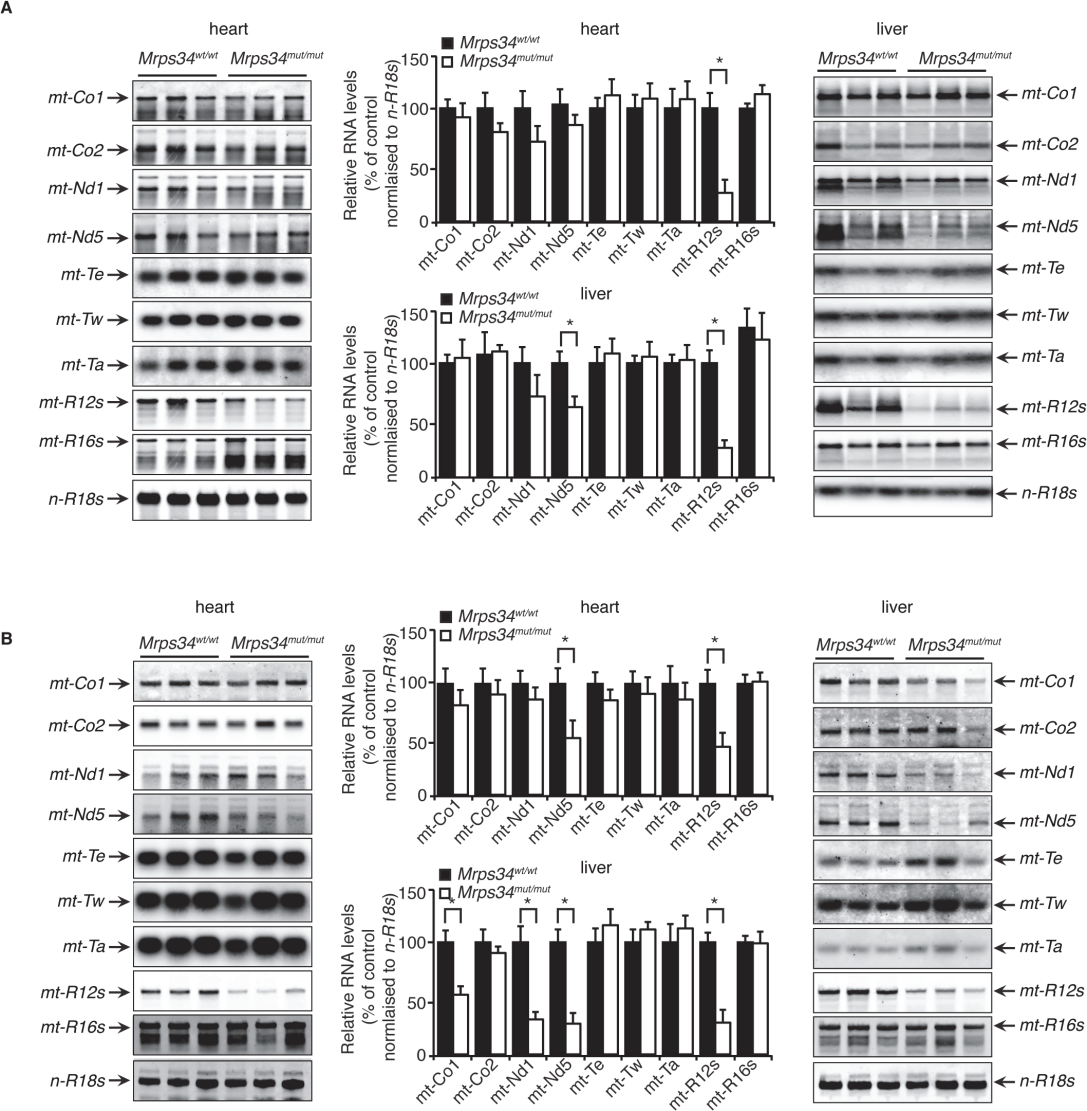
Decreased MRPS34 affects the stability of the 12S rRNA and specific mitochondrial mRNAs. (A) The abundance of mature mitochondrial transcripts in mitochondria isolated from young *Mrps34* ^*wt/wt*^ and *Mrps34* ^*mut/mut*^ livers and hearts was analyzed by northern blotting. (B) The abundance of mature mitochondrial transcripts in aged liver and heart was analyzed by northern blotting. 18S rRNA was used as a loading control. The data are representative of results obtained from at least 8 mice from each strain. Data are means ± SEM of three separate experiments; *, *p* < 0.05 compared with control treatments by a 2-tailed paired Student’s *t* test.

The mitochondrially-encoded 12S and 16S rRNAs form the scaffolds for the mitochondrial ribosomes that use 22 mitochondrial tRNAs to translate the 11 mRNAs [12]. Therefore we analyzed if the *Mrps34* mutation affected the steady-state levels and stability of mitochondrial RNAs in heart and liver by northern blotting. The 12S rRNA levels were reduced in hearts and livers of the young *Mrps34* ^*mut/mut*^ mice, and the 16S rRNA level was unaffected (Fig. 2A), suggesting that the MRPS34 is required for the stability of the 12S rRNA. In addition, we observed that the levels of the *mt-Nd5* mRNA were decreased in the livers, but not in the hearts of the young mutant mice (Fig. 2A), indicating that the *Mrps34* mutation causes tissue specific effects on mitochondrial RNA metabolism. The 12S rRNA was also reduced significantly in the heart and livers of the mutant aged mice (Fig. 2B). Furthermore, the levels of specific mitochondrial mRNAs, *mt-Co1*, *mt-Nd1* and *mt-Nd5* were decreased in the livers of aged mice. However, in the hearts of aged *Mrps34* ^*wt/wt*^ and *Mrps34* ^*mut/mut*^ mice only *mt-Nd5* was decreased but not *mt-Co1 or mt-Nd1* suggesting that in the liver the stability of specific mRNAs is more severely affected by the *Mrps34* mutation with age (Fig. 2A and 2B). The levels of mitochondrial tRNAs were unaffected in heart and liver mitochondria, suggesting that MRPS34 is necessary for the stability of 12S rRNA and for the stability of specific mRNAs.

### MRPS34 is required for mitochondrial translation

**Fig 3:**
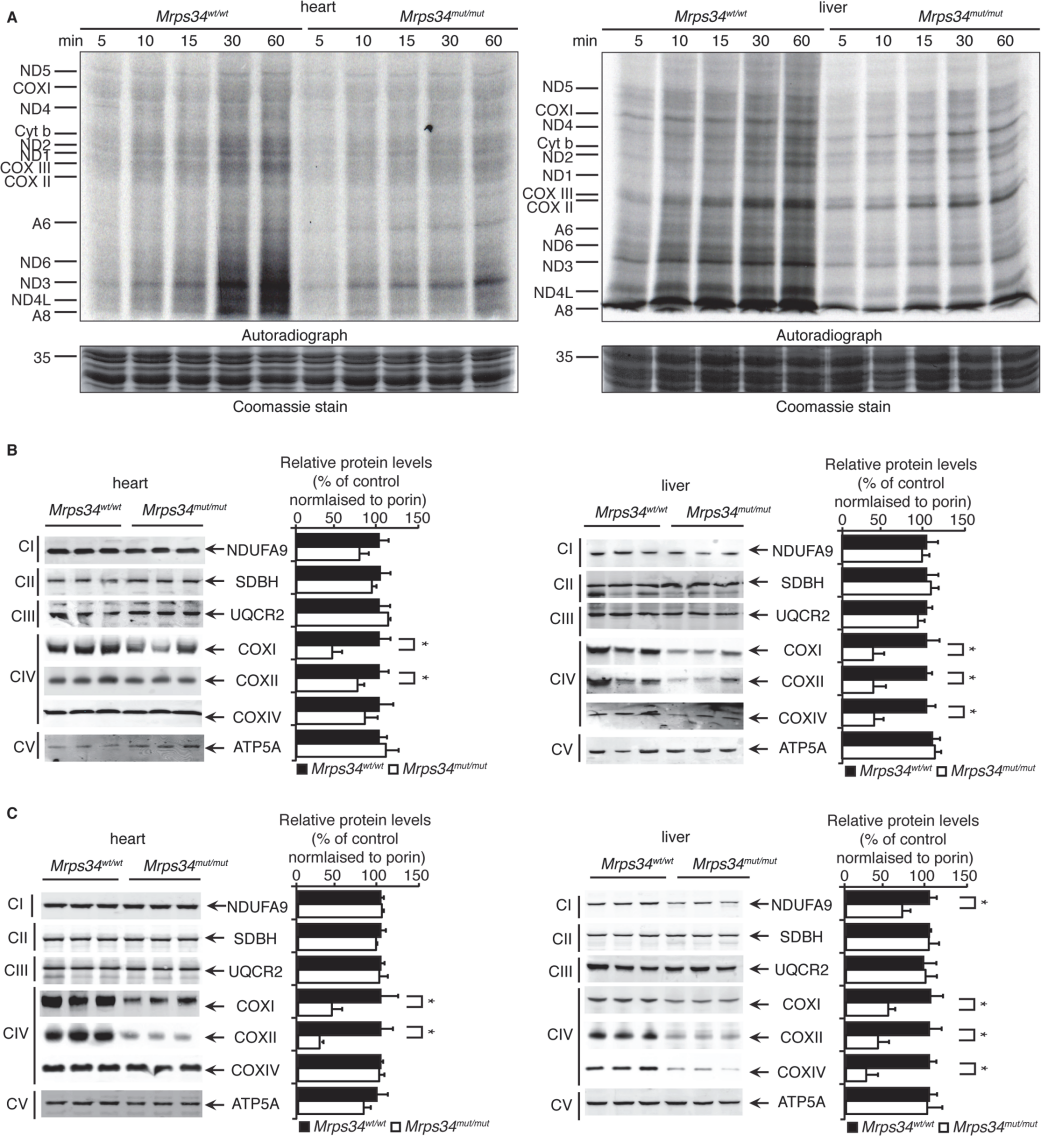
Decreased MRPS34 levels cause a reduction in mitochondrial protein synthesis and steady state levels of mitochondrial proteins. (A) Protein synthesis in heart and liver mitochondria isolated from *Mrps34* ^*wt/wt*^ and *Mrps34* ^*mut/mut*^ was measured by pulse incorporation of ^35^S-labelled methionine and cysteine over time. Equal amounts of mitochondrial lysates (50 μg) were separated by SDS–PAGE, Coomassie stained (bottom panel) and visualized by autoradiography (top panel). Mitochondrial lysates (20 μg) from *Mrps34* ^*wt/wt*^ and *Mrps34* ^*mut/mut*^ livers and hearts of young (B) and aged (C) mice were resolved by SDS-PAGE and immunoblotted using antibodies to assess changes in mitochondrial protein abundance. Porin was used as a loading control. Data are means ± SEM of four separate experiments; *, *p* < 0.05 compared with control treatments by a 2-tailed paired Student’s *t* test.

Since MRPS34 is a ribosomal protein we analyzed how decreased levels of this protein, as a result of the *Mrps34* mutation, affect translation by measuring *de novo* protein synthesis of the 13 mitochondrially-encoded polypeptides in heart and liver mitochondria from *Mrps34* ^*mut/mut*^ and *Mrps34* ^*wt/wt*^ mice. The overall decrease of mitochondrial protein synthesis in the young and aged mutant mice compared to controls (S2 Fig) indicates that MRPS34 is required for mitochondrial protein synthesis. To investigate the effects of the mutation on the rate of protein synthesis between heart and liver mitochondria we measured mitochondrial translation over time. Interestingly we observed that the initial rate of translation in control liver mitochondria is faster compared to that of heart mitochondria (Figs. 3A, S2). In addition, we observed that the *Mrps34* mutation causes a decrease in mitochondrial protein synthesis (Fig. 3A), however the initial rate in translation may account for the severity of the molecular changes found in the liver compared to heart mitochondria.

Next we analyzed the effects of the *Mrps34* mutation on the steady-state abundance of mitochondrial proteins by immunoblotting. The levels of the mitochondrially-encoded COXI and COXII were reduced in the livers and hearts of young *Mrps34* ^*mut/mut*^ mice compared to controls (Fig. 3C). In the aged mutant mice we observed a more pronounced decrease in COXI and COXII in both the heart and liver, suggesting that the effects of decreased MRPS34 levels on the steady state levels of these proteins are cumulative and the molecular defects have a late onset in the heart (Fig. 3D). In addition, we observed reduction in nuclear-encoded mitochondrial proteins, such as NDUFA9 and COXIV, possibly in a retrograde response to the decreased levels of mitochondrially-encoded proteins in the livers of aged *Mrps34* ^*mut/mut*^ mice (Fig. 3D).

**Fig 4:**
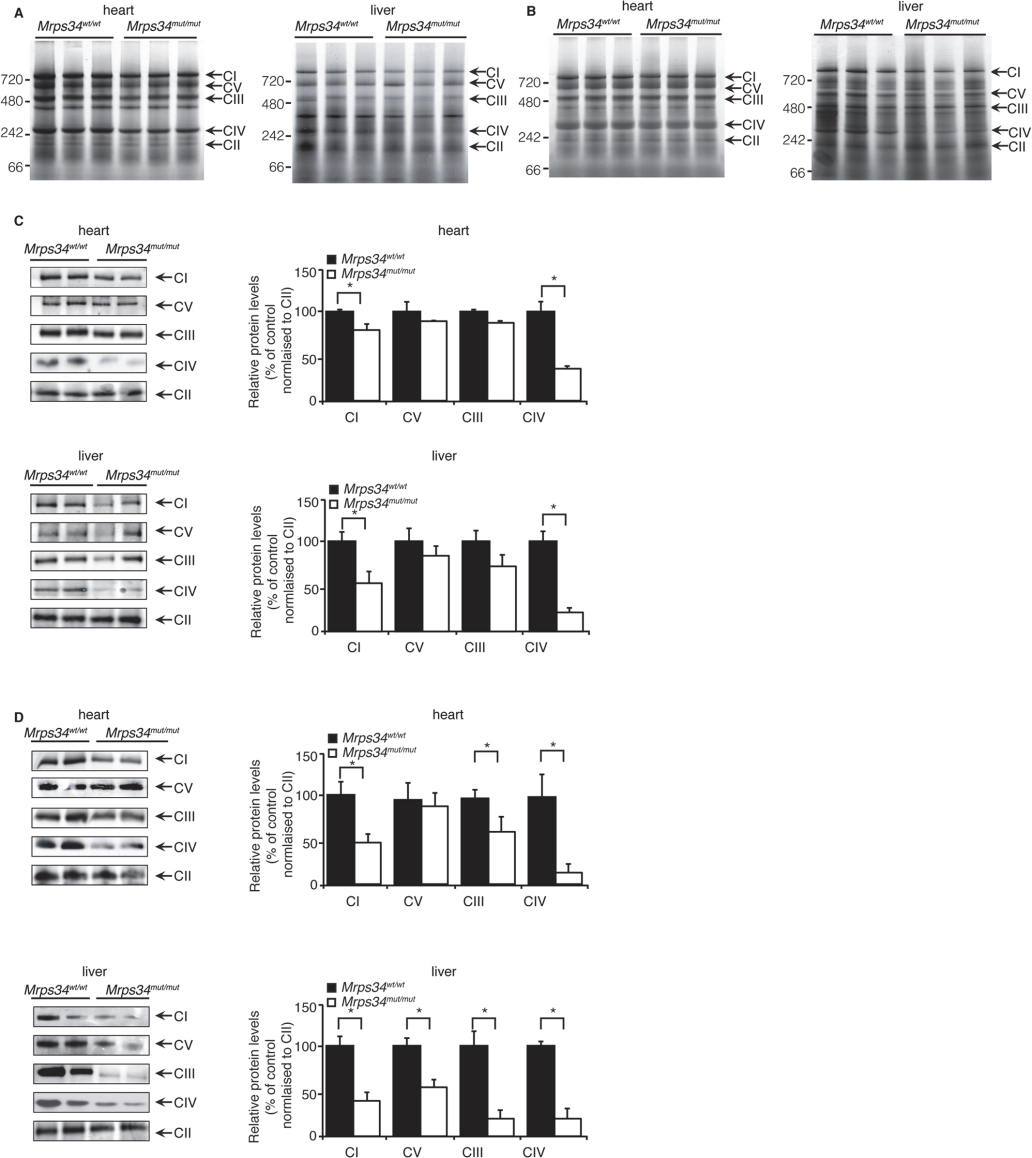
Reduced MRPS34 affects the abundance of mitochondrial respiratory complexes. BN-PAGE was performed on 75 μg of mitochondria isolated from liver and heart of young (A) and aged (B) *Mrps34* ^*wt/wt*^ and *Mrps34* ^*mut/mut*^ mice. Mitochondria lysates (75 μg) isolated from liver and heart of *Mrps34* ^*wt/wt*^ and *Mrps34* ^*mut/mut*^ young mice (C) and aged mice (D) were analyzed by BN-PAGE and the gels used for immunoblotting. Specific antibodies representing proteins of each of the mitochondrial complexes were used to compare abundance of protein in the wild type and mutant mice. Data are means ± SEM of five separate experiments; *, *p* < 0.05 compared with control treatments by a 2-tailed paired Student’s *t* test.

We investigated the effects of *Mrps34* mutation on the abundance of the mitochondrial respiratory complexes by blue native polyacrylamide gel electrophoresis (BN-PAGE). The reduction in the abundance of the respiratory complexes in hearts of *Mrps34* ^*mut/mut*^ mice is more apparent in the aged mice compared to the young (Fig. 4A and 4B). The reduction of the respiratory complexes was more significant in the livers of *Mrps34* ^*mut/mut*^ compared to *Mrps34* ^*wt/wt*^ mice (Fig. 4A and 4B) and this was confirmed by immunoblotting of each complex following BN-PAGE (Fig. 4C and 4D). Complexes I and IV were reduced in the hearts of both young and aged mice, whereas in the livers, Complexes I, III, IV and V were reduced (Fig. 4C and 4D). Taken together these findings provide evidence that MRPS34 is required for protein synthesis and that decreased mitochondrial translation as a result of the *Mrps34* mutation can have varied effects on the abundance of mitochondrial proteins and respiratory complexes in different tissues.

### The mutation in the *Mrps34* gene leads to decreased mitochondrial respiration and complex activity

**Fig 5:**
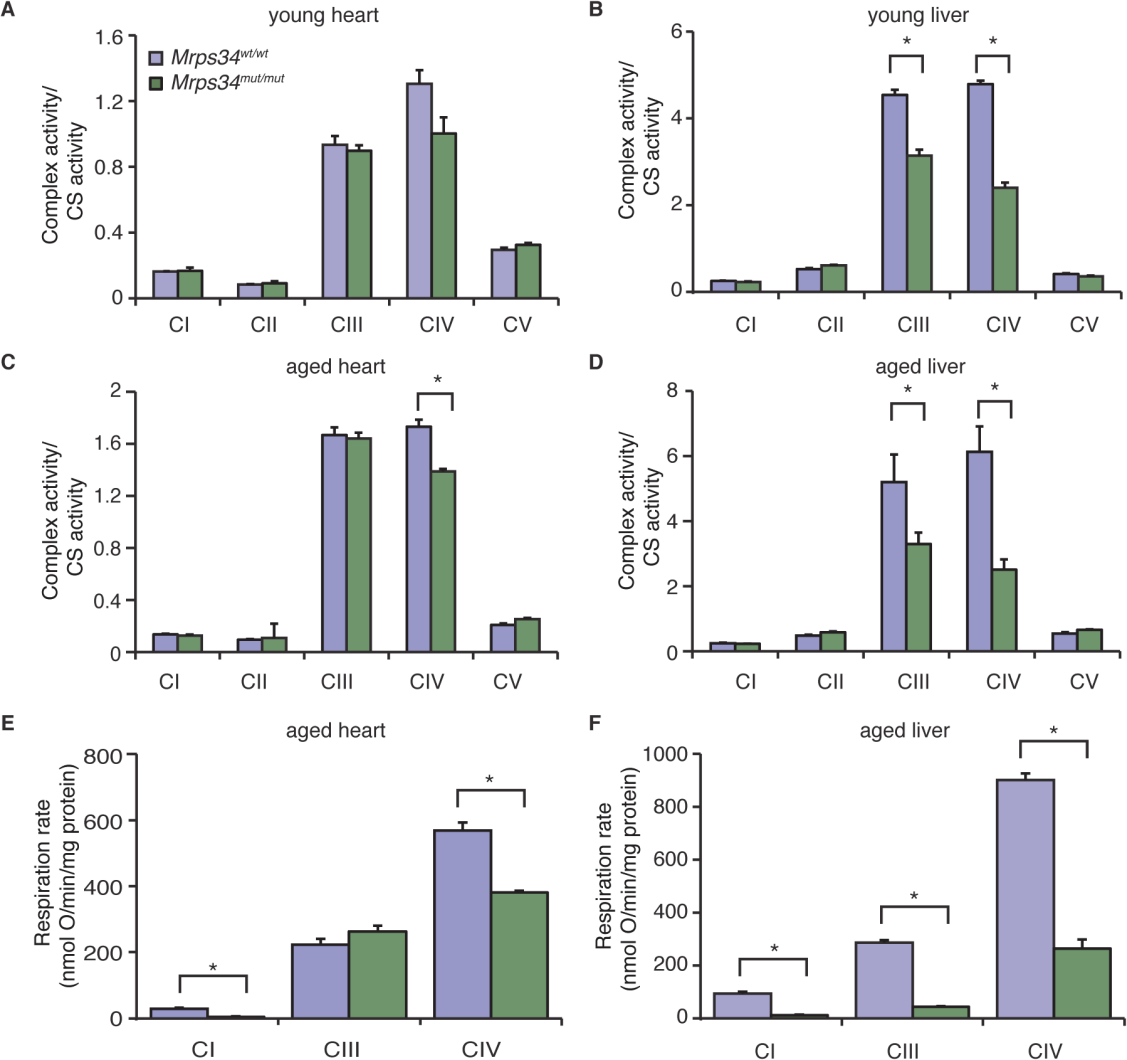
The *Mrps34* mutation causes reduced oxygen consumption and respiratory complex activities in heart and liver mitochondria. The activities of the five mitochondrial respiratory complexes were measured in mitochondria isolated from heart (A) and liver (B) of young *Mrps34* ^*wt/wt*^ and *Mrps34* ^*mut/mut*^ mice and aged mice (C and D), respectively. The respiratory complex activities were normalized relative to citrate synthase activity. Data are means ± SEM of three-four separate experiments; *, *p* < 0.05 compared with control treatments by a 2-tailed paired Student’s *t* test. State 3 and 4 respiration was measured in mitochondria isolated from hearts (E) and livers (F) of aged *Mrps34* ^*wt/wt*^ and *Mrps34* ^*mut/mut*^ mice using an OROBOROS oxygen electrode. Data are means ± SEM of three-four separate experiments; *, *p* < 0.05 compared with control treatments by a 2-tailed paired Student’s *t* test.

Because we observed a decrease in the abundance of mitochondrial respiratory complexes we measured their enzyme activities in heart and liver mitochondria from young and aged *Mrps34* ^*mut/mut*^ and *Mrps34* ^*wt/wt*^ mice. The activities of the respiratory complexes were not significantly decreased in hearts of young *Mrps34* ^*mut/mut*^ mice (Fig. 5A), although there was a trend towards a decrease in in the activity of Complex IV, likely as a result of its decreased abundance (Fig. 4C). The activities of Complexes III and IV were significantly decreased in livers of the young mutant mice (Fig. 5B), consistent with the observed decrease in the levels of these complexes relative to those in control mice. In the hearts of aged *Mrps34* ^*mut/mut*^ mice only the activity of Complex IV was decreased (Fig. 5C), whereas the activities of both Complex III and IV were most significantly affected in the livers of these mice (Fig. 5D) as a result of reduction in the abundance of these complexes identified by immunoblotting (Fig. 4C and 4D). Measurements of oxygen consumption confirmed that mitochondrial respiratory function was affected more in the livers than hearts of young *Mrps34* ^*mut/mut*^ mice and this reduction was more dramatic with age in both tissues (Figs. 5E, 5F, and S3). Consistent with the observed molecular changes in response to the *Mrps34* mutation, mitochondrial dysfunction was more pronounced in the liver compared to the heart of *Mrps34* ^*mut/mut*^ mice (Fig. 5E), suggesting that these two organs have different capacities to cope with changes in translational efficiency at different ages as observed when we measured mitochondrial translation (Fig. 3A).

### *Mrps34* ^*mut/mut*^ mice develop heart hypertrophy and liver steatosis with age

**Fig 6:**
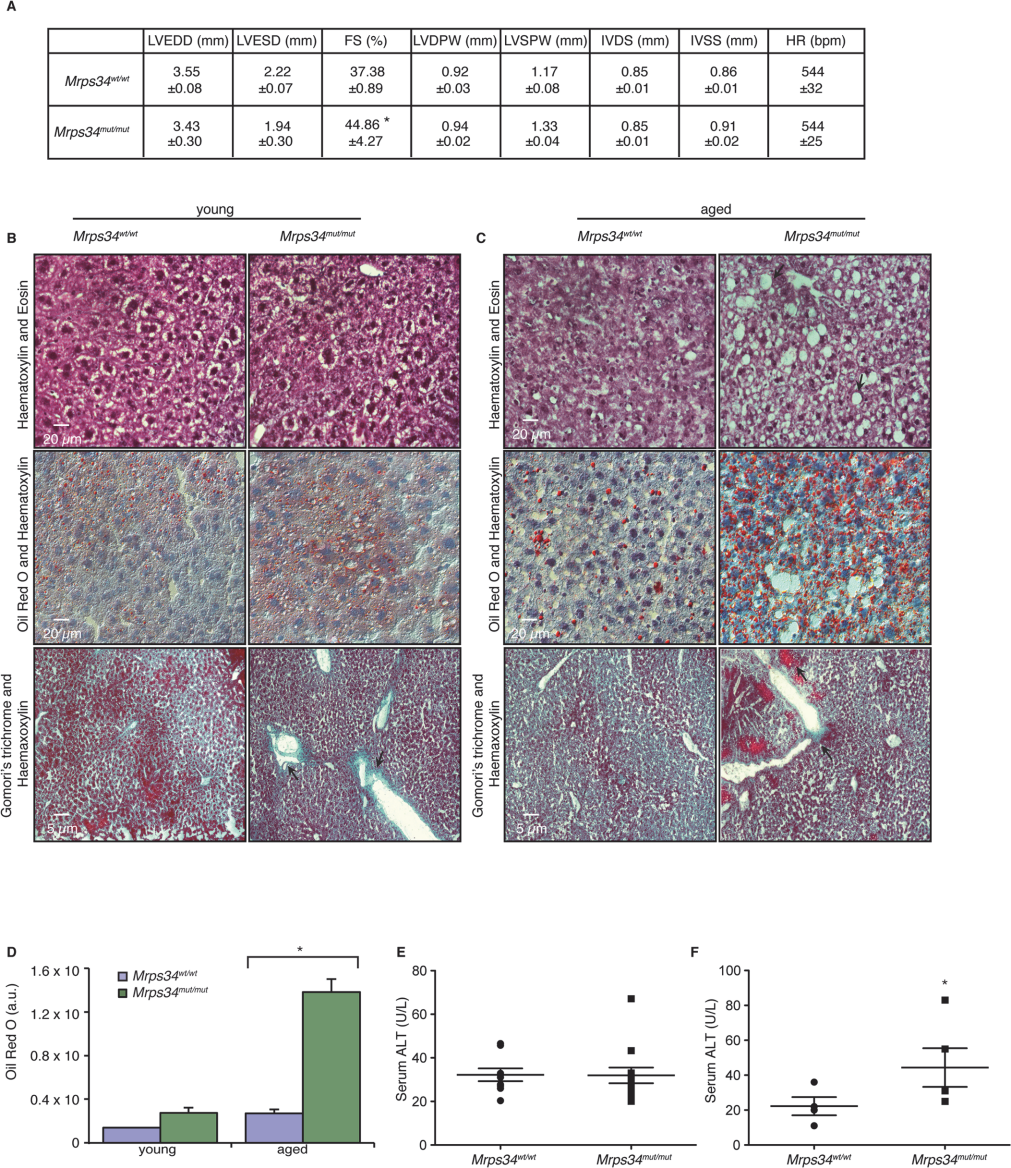
*Mrps34* ^*mut/mut*^ mice have hypertrophic hearts and increased lipid accumulation in their livers. (A) Echocardiographic parameters of *Mrps34* ^*wt/wt*^ (n = 5) and *Mrps34* ^*mut/mut*^ (n = 5) mice. LVEDD, left ventricular end diastolic diameter; LVESD, left ventricular end systolic diameter; FS, fractional shortening; LVDPW, left ventricular posterior wall in diastole; LVSPW, left ventricular posterior wall in systole; IVDS, intraventricular septum in diastole; IVSS, intraventricular septum in systole. Values are means ± standard error. *p<0.05 compared with *Mrps34* ^*wt/wt*^. Liver sections cut at 8–12 μm thickness were stained with Haematoxylin and Eosin, oil red O and Haematoxylin or Gomori trichrome from young (B) and aged (C) *Mrps34* ^*wt/wt*^ (n = 9) and *Mrps34* ^*mut/mut*^ (n = 9) mice and visualized at 40X magnification. (D) Quantitative measurement of oil red staining using Image J. Data are means ± SEM of four different mice; *, *p* < 0.05 compared with control treatments by a 2-tailed paired Student’s *t* test. Serum ALT levels in young (E) and aged (F) *Mrps34* ^*wt/wt*^ (n = 12) and *Mrps34* ^*mut/mut*^ (n = 12) mice.

Mutations in genes encoding mitochondrial ribosomal proteins have been shown to cause cardiomyopathy in patients that result in impaired mitochondrial protein synthesis and consequently reduction in the levels and activities of respiratory complexes [18,19]. The *Mrps34* ^*mut/mut*^ mice appeared similar to their *Mrps34* ^*wt/wt*^ mice littermates at birth and no developmental or fertility differences were observed compared to the control mice. However, with age the *Mrps34* ^*mut/mut*^ mice develop physiological changes that affect multiple tissues to varying extents. We observed slight reduction in vision in the *Mrps34* ^*mut/mut*^ compared to the *Mrps34* ^*wt/wt*^ mice, measured using optokinetic drum experiments, although the optic nerve was not affected (S4A–S4C, S4H Fig). The motor coordination, strength, and balance between the *Mrps34* ^*wt/wt*^ and *Mrps34* ^*mut/mut*^ mice was not significantly different (S4D–S4G Fig), although a slight difference at day 4 may suggest a potential motor learning deficit. We observed centralization of nuclei in the muscle of aged *Mrps34* ^*mut/mut*^ mice and decreased COX activity (S4I Fig). To investigate if reduced Complex IV activity, as a result of the *Mrps34* mutation, affects heart function we carried out echocardiography on the *Mrps34* ^*mut/mut*^ and *Mrps34* ^*wt/wt*^ mice. We found that the hearts of mutant mice have increased fractional shortening, a thickening (hypertrophy) of the posterior wall and associated decreased oxygen consumption (Fig. 6A) that can be a common consequence of mitochondrial dysfunction. However, because we found greater mitochondrial dysfunction in the livers of *Mrps34* ^*mut/mut*^ mice we also investigated the effects of the mutation on the morphology and function of the livers in these mice compared to *Mrps34* ^*wt/wt*^ mice. Morphological examination revealed increased lipid accumulation in the livers of the young mutant mice and this was more pronounced in the aged mutant mice, suggesting that they have developed liver steatosis (Fig. 6B). Oil red O staining revealed extensive accumulation of lipid droplets in the livers of *Mrps34* ^*mut/mut*^ mice that was significantly exacerbated with age (Fig. 6C and 6D) and correlated with increased levels of alanine aminotransferase (ALT) in the serum of these mice (Fig. 6E and 6F), which is a marker of liver dysfunction, commonly associated with liver steatosis [20]. Extensive liver dysfunction can cause fibrosis, therefore we used Gomori’s trichome to stain the livers of *Mrps34* ^*mut/mut*^ and *Mrps34* ^*wt/wt*^ mice (Fig. 6B and 6C). In the young *Mrps34* ^*mut/mut*^ mice infiltration of collagen was found around portal tracts (Fig. 6B, arrows) compared to control mice, that was more pronounced in the aged *Mrps34* ^*mut/mut*^ mice, disrupting the morphology of the liver, encapsulating the tissue (arrow) and forming nodules that are markers of liver fibrosis (Fig. 6C). Our findings indicate that the *Mrps34* mutation causes mitochondrial dysfunction that can affect multiple tissues, making these mice a model system to investigate how nuclear mutations can cause pathology with varying severity in different tissues.

### MRPS34 is required for the stability of the small ribosomal subunit and its association with the large subunit

**Fig 7:**
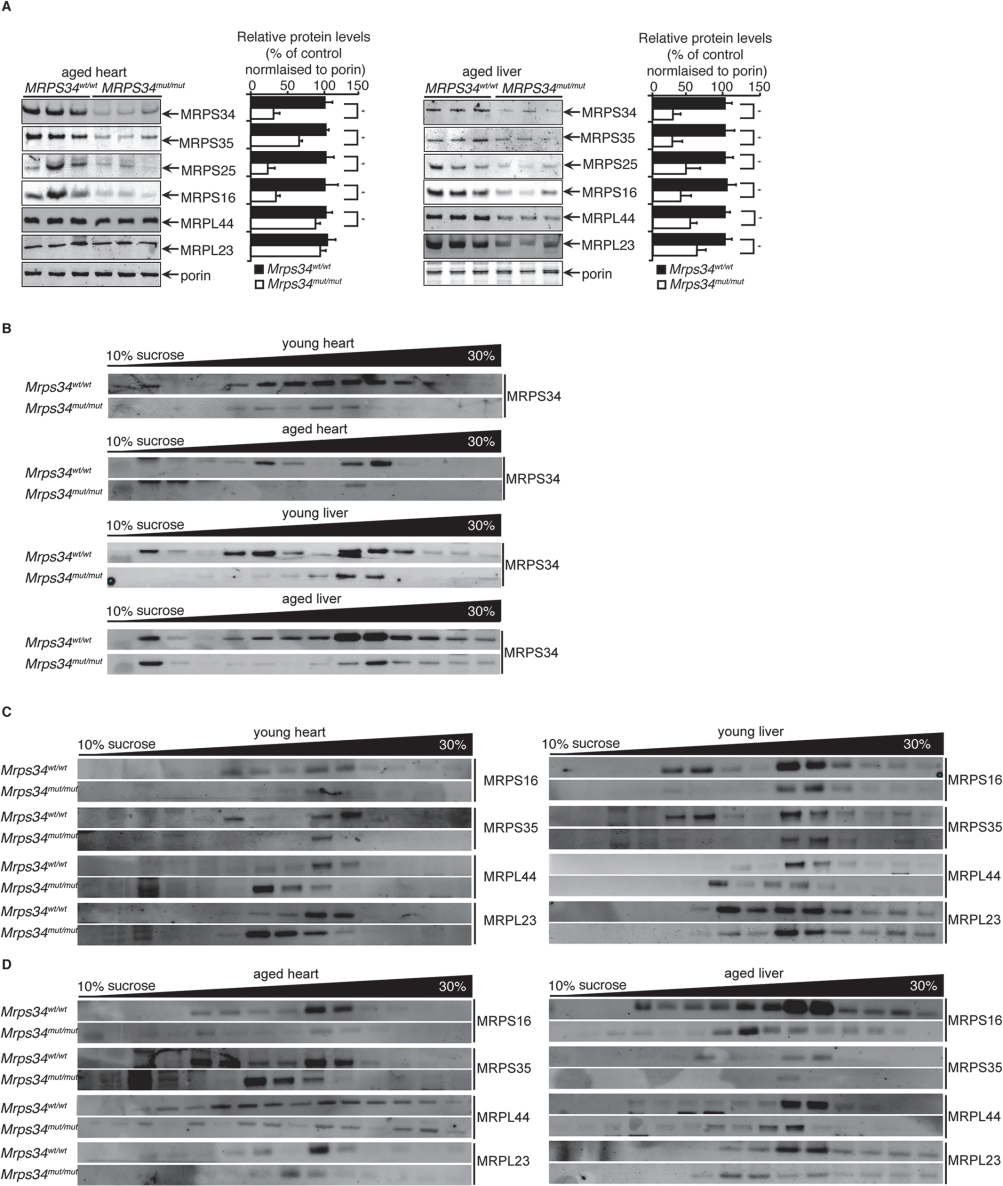
MRPS34 is required for the small ribosomal subunit and the stability of the mitoribosome. (A) Mitochondrial ribosomal protein levels in mitochondria (50 μg) isolated from liver and heart of *Mrps34* ^*wt/wt*^ compared to *Mrps34* ^*mut/mut*^ mice was determined by immunoblotting. (B) Distribution of the MRPS34 protein in 10–30% sucrose gradients of heart (0.8 mg) and liver (1.2 mg) mitochondria from young and aged *Mrps34* ^*wt/wt*^ and *Mrps34* ^*mut/mut*^ mice. Mitochondrial protein lysates from heart (0.8 mg) and liver (1.2 mg) of young (C) and aged (D) *Mrps34* ^*wt/wt*^ and *Mrps34* ^*mut/mut*^ were fractionated on 10–30% sucrose gradients. The distribution of ribosomal proteins was analyzed by immunoblotting against antibodies that are markers of the small and large ribosomal subunits. The data are typical of results from at least three independent biological experiments.

To understand the role of MRPS34 within the mitochondrial ribosome we investigated the effects of the *Mrps34* mutation on the levels of mitochondrial ribosomal proteins from the small and large subunit in *Mrps34* ^*mut/mut*^ compared to *Mrps34* ^*wt/wt*^ mice by immunoblotting. It has previously been shown that the loss of certain mitochondrial small ribosomal subunit proteins can disrupt the assembly of the small subunit and cause the loss or reduction of other small ribosomal subunit proteins [21], while loss of other small subunit proteins does not [22], potentially revealing their roles in ribosome assembly. We investigated the levels of MRPS16, MRPS25, and MRPS35 mitochondrial small ribosomal subunit proteins, and found that their levels closely paralleled those of MRPS34 (Fig. 7A), suggesting that the *Mrps34* mutation causes destabilization of the small ribosomal subunit. This reduction in the small ribosomal subunit proteins is consistent with the reduced levels of the 12S rRNA (Fig. 2A and 2B). Although in the young mutant mice the levels of the large ribosomal subunit protein MRPL44 were slightly increased by the *Mrps34* mutation (Fig. 1C), likely as a compensatory response to decreased small ribosomal subunit proteins (as previously observed in [23]), we observed that in the aged mutant mice the MRPL44 and MRPL23 proteins were also decreased compared to controls (Fig. 7A) both in heart and liver mitochondria. As ribosomal proteins are required in tightly regulated ratios, decrease in MRPS34 over time leads to reduction in other mitoribosomal proteins. Therefore we conclude that MRPS34 is required for the steady-state levels of small subunit ribosomal proteins, and thereby the stability of the small ribosomal subunit, which is necessary for decoding of mitochondrial mRNAs when coupled with the large subunit and consequently translation of the mitochondrially-encoded proteins.

Next we investigated if the residual levels of the mutant MRPS34 protein can interact with the mitochondrial ribosome using sucrose gradients followed by immunoblotting for this protein in heart and liver mitochondrial from the mutant and control mice (Fig. 7B). We observed that there are significantly reduced levels of the MRPS34 protein in both heart and liver mitochondria, however the mutant protein can associate with the small ribosomal subunit and constitute part of the actively translating mitoribosome.

To determine if MRPS34 is required for the stability of the mitoribosome we analyzed the mitochondrial ribosome profile of the large and small subunit, the monosome and polysome on sucrose gradients followed by immunoblotting of ribosomal marker proteins (Fig. 7C and 7D). In heart and liver mitochondria from young *Mrps34* ^*mut/mut*^ mice we observed a decrease in the presence of the small ribosomal proteins MRPS16 and MRPS35 and consequently decrease in the presence of actively translating mitochondrial ribosomes. Similarly, we observed a decrease in the actively translating mitochondrial ribosome in young heart and liver mitochondria when we immunoblotted for the large ribosomal subunit markers MRPL44 and MRPL23 and a small increase in the presence of the large subunit consistent with the slight increase in the steady state levels of these proteins (Fig. 1C), that are more apparent in heart mitochondria (Fig. 7C). The decreased levels of the small ribosomal subunit result in decreased formation of mitochondrial ribosomes.

In liver and heart mitochondria from aged mutant mice we observe decreased levels in both the small and the large ribosomal subunits and consequently reduced levels of the actively translating mitoribosomes (Fig. 7D). In the aged *Mrps34* ^*mut/mut*^ mice we found that both the ribosomal proteins from small and large mitochondrial subunits are redistributed compared to control mice and we observe reduced polysome formation in the mutant mice, indicating destabilization of mitoribosomes and polysomes with age (Fig. 7D), likely as a result of decreased MRPS34 levels (Fig. 7A and 7B). These results indicate that reduced abundance of the MRPS34 protein affects the rate of translation through destabilization of the small ribosomal subunit and the 12S rRNA that are required for mitoribosome formation.

## Discussion

The mitochondrial ribosome is a unique molecular machine that has been honed through evolution to cope with the compaction of the mitochondrial genome [24]. The lack of substantial untranslated regions has given rise to leaderless mitochondrial mRNAs that are somehow recognized by the mitoribosome for translation. These ribosomes have acquired additional mitochondria-specific proteins that predominantly decorate the surface of the ribosome, giving clues that these proteins may play a role in specific recognition and recruitment of mRNAs to the mitoribosome, modulation of translation according to mitochondrial environmental changes, and the exclusive translation of hydrophobic membrane proteins [7,10]. The mitochondria-specific proteins are largely uncharacterized and understanding their roles in the mitoribosome would provide insight into the unique features of mitochondrial protein synthesis that are different from cytoplasmic and prokaryotic systems. The MRPS34 protein was identified as a constituent of the small ribosomal subunit [6,25], it shares little homology to other proteins and is conserved from humans to zebrafish. Here we show that a point mutation in the mouse *Mrps34* gene, causing a leucine to proline change in a conserved alpha helix, results in a significant reduction of the MRPS34 protein in heart and liver mitochondria. Consequently, mitochondrial protein synthesis is impaired in the mice homozygous for the *Mrps34* mutation leading to pathologies with varying severities in different tissues.

Decreased levels of MRPS34 affect the stability of mitochondrial RNAs and this is particularly pronounced in the liver. The 12S rRNA is specifically decreased in the hearts and livers of the mutant mice, although this reduction is more profound in the liver, particularly with age, indicating that MRPS34 is required for the stability of the 12S rRNA. Although the mutation causes significant reduction of MRPS34, the remaining protein is assembled within the small subunit of the mitoribosome. However the significant decrease in 12S rRNA levels as a result of the MRPS34 reduction suggests that this protein may be required early in the assembly of the small ribosomal subunit and is likely a 12S rRNA-binding protein. We found that reduction in MRPS34 levels affects the abundance of small ribosomal subunit proteins. In the young mice the proteins of the large ribosomal subunit were present in substantial amounts, suggesting that the large subunit was still fully assembled despite the significant loss of the small subunit. This is further corroborated by the reduction in abundance of the 12S rRNA, which is readily degraded unless incorporated into a ribosomal subunit [26], and the uninterrupted presence of the 16S rRNA. This finding suggests that there is no regulatory cross-talk monitoring the levels of the mitochondrial large and small subunit proteins, instead there seems to be a compensatory mechanism that increases the level of large ribosomal proteins in response to loss of small ribosomal proteins. However this may not be long lasting as with age persistent loss of the small ribosomal subunit also leads to reduction of the large ribosomal subunit proteins and destabilization of the actively translating ribosomes in the *Mrps34* ^*mut/mut*^ mice. In the mutant mice we observed re-distribution of mitochondrial ribosomal proteins suggesting that the assembly of mitochondrial ribosomes is compromised when there are insufficient levels of MRPS34. The differential distribution of the ribosomal subunits in the mutant compared to control mice suggests that there is accumulation of ribosome assembly intermediates due to the reduced MRPS34 levels. In the young mice this re-distribution is observed solely in the small ribosomal subunit proteins indicating that the small ribosomal subunit is dissociating while the large remains stable. This may result in the formation of inaccurately assembled ribosomes, which are easily dissociated into subunits or are unable to bind to docking sites on the inner mitochondrial membrane (IMM) affecting protein synthesis and OXPHOS biogenesis. As a result of this we find that over time, in the aged mice, the stability of the mitochondrial ribosome is affected causing a reduction in polysome formation. These changes may account for the more obvious mitochondrial dysfunction in the aged mutant mice. Furthermore, as the ribosomal proteins are required in a particular stoichiometry, loss of a ribosomal protein would destabilize this balance and would result in decreased assembly of *de novo* mitochondrial ribosomes. This is confirmed by our data where persistent decrease in MRPS34 in the aged mutant mice leads to reduction of actively translating ribosomes as a result of a decrease in small and large ribosomal subunit proteins.

We found decreased levels of specific mRNAs, including *mt-Nd1*, *mt-Nd5* and *mt-Co1* in the livers of mutant mice, while only the *mt-Nd5* mRNA was reduced in the hearts of aged mutant mice. The levels of other mRNAs such as *mt-Co2* and the tRNAs were unaffected in both heart and liver, and with age. Taken together these findings reveal that MRPS34 is necessary for the stability of specific mitochondrial mRNAs and may indicate that the active translation of certain mRNAs is linked to their stabilities. The different effects on the mitochondrial mRNAs between heart and liver reflect tissue-specific changes that have been found in mitochondrial disease patients previously [18,27,28], as well as recently in a mouse model of mitochondrial disease [29]. In the heart, mitochondrial RNAs account for at least 30% of the total RNA [30] reflecting the dependence of the heart on OXPHOS. It is likely that in the heart there is excess production of mitochondrial mRNAs relative to the required threshold for normal function, such that decreases in mRNA levels do not compromise mitochondrial function in the short term. This is not the case in the liver where the decrease in mitochondrial RNAs has more profound effects on energy metabolism. Furthermore we found that MRPS34 is more abundant in the heart compared to the liver of control mice, which may protect the heart from a more pronounced decline in function in the mutant mice. We find that the levels of the other mitoribosomal proteins in control mice is higher in the heart compared to the liver, likely due to the higher mitochondrial content in the heart. Finally we observe that the liver has a faster initial rate of mitochondrial translation that is compromised in the mutant mice and may contribute to the more pronounced defect in the liver compared to the heart. It could be that after the initial burst of translation in the liver the levels of mitoribosomes or their recycling become limiting so that subsequently translation plateaus, whereas in the heart the higher abundance of mitoribosomes contributes to the steady rate of translation. Unlike the heart, the liver is highly proliferative and it may require rapid bursts of mitochondrial translation for its normal function and during regeneration.

Mitochondrial diseases caused by mutations in nuclear genes encoding mitochondrial proteins can affect the function of many different tissues with varying severity (reviewed in [2,3]). Furthermore, nuclear mutations in mitochondrial proteins can result in remarkably heterogeneous defects with varying severity in different tissues that are poorly understood currently [2]. We observed similar varying defects in different tissues in the *Mrps34* ^*mut/mut*^ mice, which have a fractional shortening of their hearts, centralized nuclei in their muscles and increased lipid accumulation in their livers that causes liver steatosis. Hepatopathies have been identified along with a range of different symptoms in many mitochondrial disorders, although hepatopathy was the main consequence of a mutation in the tRNA 5-methylaminomethyl-2-thiouridylate methyltransferase (TRMU) gene that produces the enzyme responsible for 2-thiouridylation of the tRNA^Glu^, tRNA^Gln^ and tRNA^Lys^, causing a severe but reversible infantile hepatopathy [31–33]; the mutation in the *Mrps34* gene is the first to show pronounced liver defects in mice providing the means to investigate the contribution of mitochondrial function to hepathopathy in the future. Many mutations in nuclear genes that encode protein components of the translational machinery result in compromised biogenesis of specific or all mitochondrial respiratory chain complexes and lead to decreased OXPHOS and some of these have been shown to cause accumulation of lipids in hepatocytes [3,34]. Although we observe overall decrease in mitochondrial protein synthesis as a result of the *Mrps34* mutation, the greatest reduction is in the activity of Complex IV in both heart and liver, and this reduction is greater in the liver. Decrease in the *mt-Co1* mRNA and consequently the COXI protein, that is necessary for the biogenesis of Complex IV [35] likely contributes to this pronounced decrease in its abundance and activity.

Our work has shown that MRPS34 plays a role in maintaining the stability of the small ribosomal subunit and the 12S rRNA, which are necessary formation of actively translating mitoribosomes. In addition, MRPS34 is required for the stability of specific mRNAs, indicating that the mitochondria-specific ribosomal proteins might have unique roles in mitochondrial RNA metabolism. Because the *Mrps34* mutation in mice is not embryonic lethal it has provided a means to investigate how mitochondrial dysfunction can lead to disease in the whole body and the progression of the disease with age. Establishment of mouse models where mitochondrial dysfunction causes disease are particularly important for understanding the causes of human disease pathology, identification of drug targets and for the development of future treatments for these diseases.

## Methods

### Animals and housing

Male age- and litter-mate matched (6–8 weeks ‘young’ and 30 weeks ‘aged’) wild-type (*Mrps34* ^*wt/wt*^) and homozygous (*Mrps34* ^*mut/mut*^) ENU mutant mice on a C57BL/6J background were obtained from the Australian Phenomics Facility. The *Mrps34* mice were bred onto a C57BL/6J background for 8–10 generations. Animals were singly housed in standard cages (45 cm × 29 cm × 12 cm) under a 12-h light/dark schedule (lights on 7 a.m. to 7 p.m.) in controlled environmental conditions of 22 + 2°C and 50 + 10% relative humidity. Normal chow diet (Rat & Mouse Chow, Speciality Foods, Glen Forrest, Western Australia) and water were provided ad libitum. The study was approved by the Animal Ethics Committee of the UWA (AEC 03/100/526) and performed in accordance with Principles of Laboratory Care (NHMRC Australian code for the care and use of animals for scientific purposes, 8^th^ Edition 2013). Behavioral and motor tests are described in Supplemental Methods (S1 Text).

### Tissue homogenate preparation

Tissues were homogenised in 100 μl of 100 mM Tris, 2 mM Na_3_VO_4,_ 100 mM NaCl, 1% Triton X-100, 1 mM EDTA, 10% Glycerol, 1 mM EGTA, 0.1% SDS, 1 mM NaF, 0.5% deoxycholate, 20 mM Na_4_P_2_O_7_, pH 7.4 containing PhosSTOP Phosphatase Inhibitor Cocktail and EDTA-free Complete protease inhibitor cocktail and the supernatant was collected after centrifugation at 10,000 *g*.

### Mitochondrial isolation

Mitochondria were isolated from homogenized hearts and livers and isolated by differential centrifugation as described previously [36] with some modifications. Livers were homogenized in buffer containing 250 mM sucrose, 5 mM Tris, 1mM EGTA, pH 7.4 with EDTA-free Complete protease inhibitor cocktail (Roche) and hearts were homogenized in 210 mM mannitol, 70 mM sucrose, 10 mM Tris, 0.1mM EDTA pH 7.4 containing EDTA-free Complete protease inhibitor cocktail.

### Sucrose gradient subfractionation

Liver (1.2 mg of protein) and heart (0.8 mg of protein) mitochondria were lysed with 1% n-Dodecyl β-D-maltoside in 10mM Tris-HCl, pH 7.4, 260 mM sucrose, 100 mM KCl, 20 mM MgCl_2_ in the presence of RNasin and protease inhibitors for 30 min, the lysate centrifuged at 10,000 *g* for 45 min at 4°C, the clarified lysate was loaded on a continuous 10–30% sucrose gradient (in 10 mM Tris-HCl, pH 7.4, 100 mM KCl, 20 mM MgCl_2_ in the presence of RNasin and protease inhibitors) and centrifuged at 71,000 *g* in an Optima Beckman Coulter preparative ultracentrifuge as described before [6]. Fractions were collected and precipitated with 30% trichloroacetic acid, washed in acetone, and the entire fraction was resolved by SDS-PAGE. Protein markers of the mitochondrial ribosomal subunits were detected by immunoblotting as described below.

### RNA isolation and northern blotting

RNA was isolated from heart and liver mitochondria using the miRNeasy Mini kit (Qiagen) incorporating an on-column RNase-free DNase digestion to remove all DNA. RNA (5 μg) was resolved on 1.2% agarose formaldehyde gels, then transferred to 0.45 μm Hybond-N^+^ nitrocellulose membrane (GE Lifesciences) and hybridized with biotinylated oligonucleotide probes specific to mouse mitochondrial tRNAs, mRNAs and rRNAs. The hybridizations were carried out overnight at 50°C in 5x SSC, 20 mM Na_2_HPO_4_, 7% SDS and 100 μg.ml^-1^ heparin, followed by washing. The signal was detected using streptavidin-linked infrared antibody (diluted 1: 2,000 in 3x SSC, 5% SDS, 25 mM Na_2_HPO_4_, pH 7.5) using an Odyssey Infrared Imaging System (Li-Cor).

### Immunoblotting

Specific proteins were detected using rabbit polyclonal antibodies against: MRPL44, MRPL23, MRPS16, MRPS35, MRPS25 (Proteintech, diluted 1:1000), MRPS34 (Sigma, diluted 1:1000) and mouse monoclonal antibodies against: porin, NDUFA9, Complex II, Complex III, COXI, COXII, COXIV and Complex V subunit (Abcam, diluted 1:1000), in Odyssey Blocking Buffer (Li-Cor). IR Dye 800CW Goat Anti-Rabbit IgG or IRDye 680LT Goat Anti-Mouse IgG (Li-Cor) secondary antibodies were used and the immunoblots were visualized using an Odyssey Infrared Imaging System (Li-Cor). Tissue specific immunoblotting analysis was performed on a Proteintech mouse tissue blot (Cat. No. M10005).

### Mitochondrial protein synthesis

Mitochondrial *de novo* protein synthesis was analyzed using Expres35S Protein Labelling Mix [35S] (14 mCi, Perkin–Elmer) as described before [23].

### Blue Native Page Electrophoresis

Liver and heart mitochondrial lysates were resolved by BN-PAGE to detect the respiratory complexes by Coomassie staining as described previously [8,37] or by immunoblotting as described above.

### Complex enzyme assays

The enzyme activities of all five respiratory complexes and citrate synthase were measured in a 1 ml cuvette at 30°C using a Perkin Elmer lambda 35 dual beam spectrophotometer as described in [38].

### Respiration

Mitochondrial respiration was evaluated as O_2_ consumption in isolated heart and liver mitochondria according to Kuznetsov et al. Mitochondria were supplemented with substrates for either complex I (10 mM glutamate/malate, Sigma), II (10 mM succinate, Sigma) or III (1 mM TMPD/1 mM ascorbate, Sigma). After addition of 1 mM adenosine diphosphate (ADP, Sigma) to the recording chamber, State 3 respiration activity was measured. ADP independent respiration activity (State 4) was monitored after addition of oligomycin (2 μg/ml, Sigma).

### Echocardiography

Echocardiographic studies to measure left ventricular function were performed on mice under light methoxyflurane anesthesia with the use of an i13L probe on a Vivid 7 Dimension (GE Healthcare). Echocardiographic measurements were taken on M-mode in triplicate from each mouse and the quantitative measurements represent the average. M-mode recordings were made at a sweep speed of 200 mm/s. Measurements of left ventricular end diastolic diameter (LVEDD), left ventricular end systolic diameter (LVESD), fractional shortening (FS), left ventricular posterior wall in diastole (LVDPW), left ventricular posterior wall in systole (LVSPW), intraventricular septum in diastole (IVDS), and intraventricular septum in systole (IVSS) were made. Fractional shortening (FS) was calculated by the formula [(LVEDD-LVESD)/EDD] x 100.

### Histochemistry

Fresh sections of the liver and muscle were frozen in Optimal Cutting Temperature (OCT) medium, sectioned and stained with Haematoxylin and Eosin, Gomori’s Trichrome and Haematoxylin, Oil Red O and Haematoxylin, COX or NADH stains. Images were acquired using a Nikon Ti Eclipse inverted microscope using a Nikon 40x objective and Oil Red O staining was quantified as described previously [39].

## Supporting Information

S1 Text Supplemental Methods. Behavioral and motor testing.
(DOC)

S1 Fig MRPS34 protein levels in mouse tissues. The distribution of MRPS34 and β-actin in tissue homogenates from *Mrps34* ^*wt/wt*^ and *Mrps34* ^*mut/mut*^ mice was determined by immunoblotting.
(TIF)

S2 Fig Decreased MRPS34 levels cause a reduction in mitochondrial protein synthesis. Protein synthesis of mitochondrially encoded proteins was measured in heart and liver mitochondria from young (A) and old (B) *Mrps34* ^*wt/wt*^ (n = 5) and *Mrps34* ^*mut/mut*^ (n = 5) by pulse incorporation of ^35^S-labeled methionine and cysteine after 1 hour. Equal amount of cell lysate protein (50 μg) was separated on SDS polyacrylamide gels and stained with Coomassie blue. The data shown are representative results of at least five different experiments. (C) Quantification of the rate of translation measured by pulse incorporation of ^35^S-labelled methionine and cysteine over time of COXIII and ND3 in heart and liver mitochondria from aged mice. Data are means ± SEM of three separate experiments.
(TIF)

S3 Fig The *Mrps34* mutation causes reduced oxygen consumption in heart and liver mitochondria of young mice. State 3 and 4 respiration was measured in mitochondria isolated from hearts and livers of young *Mrps34* ^*wt/wt*^ and *Mrps34* ^*mut/mut*^ mice using an OROBOROS oxygen electrode. Data are means ± SEM of three separate experiments; *, *p* < 0.05 compared with control treatments by a 2-tailed paired Student’s *t* test.
(TIF)

S4 Fig Physiological effects of the *Mrps34* mutation. (A) Comparison of *Mrps34* ^*wt/wt*^ (n = 5) and *Mrps34* ^*mut/mut*^ (n = 5) tracking ability using optokinetic drum, measured in number of time spent tracking in seconds. (B) Comparison of *Mrps34* ^*wt/wt*^ and *Mrps34* ^*mut/mut*^ tracking ability measured in number of tracks performed. (C) Comparison of time spent in light versus dark in *Mrps34* ^*wt/wt*^ (n = 5) and *Mrps34* ^*mut/mut*^ (n = 5) mice measured in seconds. (D) Quantitation of behavioral studies evaluating number of times the box was reached in hanging wire experiments comparing *Mrps34* ^*wt/wt*^ (n = 5) and *Mrps34* ^*mut/mut*^ (n = 5) mice. (E) Quantitation of behavioral studies evaluating distance travelled along the wire in hanging wire experiments comparing *Mrps34* ^*wt/wt*^ and *Mrps34* ^*mut/mut*^ mice. (F) Rotarod results measured in seconds spent on the rotorrod over 4 days to show improvement and learning ability. (G) Time spent on the rotarod over 4 days to analyze motor function and learning ability. (H) Cresyl violet/toluidine blue staining of optic nerves from *Mrps34* ^*wt/wt*^ (n = 5) and *Mrps34* ^*mut/mut*^ (n = 5) mice visualized at 100x magnification. (I) Muscle sections cut at 10 μm thickness were stained with Haematoxylin and Eosin, COX and NADH from young and aged *Mrps34* ^*wt/wt*^ (n = 9) and *Mrps34* ^*mut/mut*^ (n = 9) mice and visualized at 40X magnification.
(TIF)
