# Supplementary material for: Mutation in MRPS34 Compromises Protein Synthesis and Causes Mitochondrial Dysfunction
Source: PLoS Genet. 2015 Mar 27;11(3):e1005089. doi: 10.1371/journal.pgen.1005089 (PMC4376678; doi:10.1371/journal.pgen.1005089)
Supplement: S1 Text — Behavioral and motor testing. (DOC) [file pgen.1005089.s001.doc]

**Supplemental Methods**

**Behavioral and motor testing**

*Light-dark exploration test*

A light-dark tunnel was used to investigate locomotor activity and light sensitivity (1). The 2 m long enclosed perspex tunnel (~ 15 cm x 15 cm square) was divided into a light (transparent) half and a dark (opaque) half. Each mouse was placed at the farthest end of the light half and the tunnel was sealed. Over the course of 10 minutes the number of times the mouse crossed from the light to the dark side was recorded, as well as the total amount of time the mouse spent in the light.

*Optomotor response*

The mouse visual response to moving stimuli was investigated using an optokinetic drum fitted with a moving stimulus pattern of black and white stripes of 1cm width (2). The drum comprised a motorised Perspex cylinder of 30 cm diameter, which rotated around a 13 cm diameter central pedestal, with a rotational speed of 2 revolutions per minute. Mice were placed in a non-reflective transparent cylindrical container on the central pedestal. After a 2 minute acclimatization period, the mouse was video recorded with the drum rotating clockwise for 2 minutes and then anticlockwise for 2 minutes, with a 2 minutes rest period in between. The video was analyzed to record the number of tracking movements and the total time spent tracking in the 2-minute period. There were not significant differences between clockwise and anticlockwise directions so an average was taken for each mouse.

*Hanging wire test*

In order to test for neuromuscular abnormalities (3) mice were placed (front paws only) in the middle of a 1 mm diameter horizontal wire 50 cm above the ground. The wire was 1m long with a wooden platform that the mice could climb onto at each end. Each mouse was tested 4 times on 4 consecutive days. On each day the time taken to complete the task and distance travelled was recorded. The task was stopped if the mouse fell off the wire (judged unsuccessful) or after a maximum of 1 minute 30 seconds if the mouse had not yet reached the platform.

*Rotarod test*

Tests for muscular coordination and strength were determined by performance on an accelerating rotarod. Mice were placed on a rotating drum that gradually accelerated from 2 rpm to 40 rpm during a 5-min test. The results of 3 replicates of each tested were averaged and repeated for daily for 4 consecutive days to measure baseline skill and motor learning ability.

**Supplemental References:**

1. Crawley, J. J., and Goodwin, F. K. F. (1980) Preliminary report of a simple animal behavior model for the anxiolytic effects of benzodiazepines. *Pharmacology Biochemistry and Behavior* **13**, 167–170. http://pubget.com/site/paper/6106204.

2. Abdeljalil, J. J., Hamid, M. M., Abdel-Mouttalib, O. O., Stéphane, R. R., Raymond, R. R., Johan, A. A., José, S. S., Pierre, C. C., and Serge, P. P. (2005) The optomotor response: A robust first-line visual screening method for mice. *Vision Research* **45**, 1439-1446. DOI:10.1016/j.visres.2004.12.015.

3. Del Bel, E. A., Souza, A. S., Guimarães, F. S., da-Silva, C. A., and Nucci-da-Silva, L. P. (2002) Motor effects of acute and chronic inhibition of nitric oxide synthesis in mice. *Psychopharmacology* **161**, 32–37. DOI: 10.1007/s00213-002-1009-2.
