# Supplementary figures and images for: Mutation in MRPS34 Compromises Protein Synthesis and Causes Mitochondrial Dysfunction
Source: PLoS Genet. 2015 Mar 27;11(3):e1005089. doi: 10.1371/journal.pgen.1005089 (PMC4376678; doi:10.1371/journal.pgen.1005089)

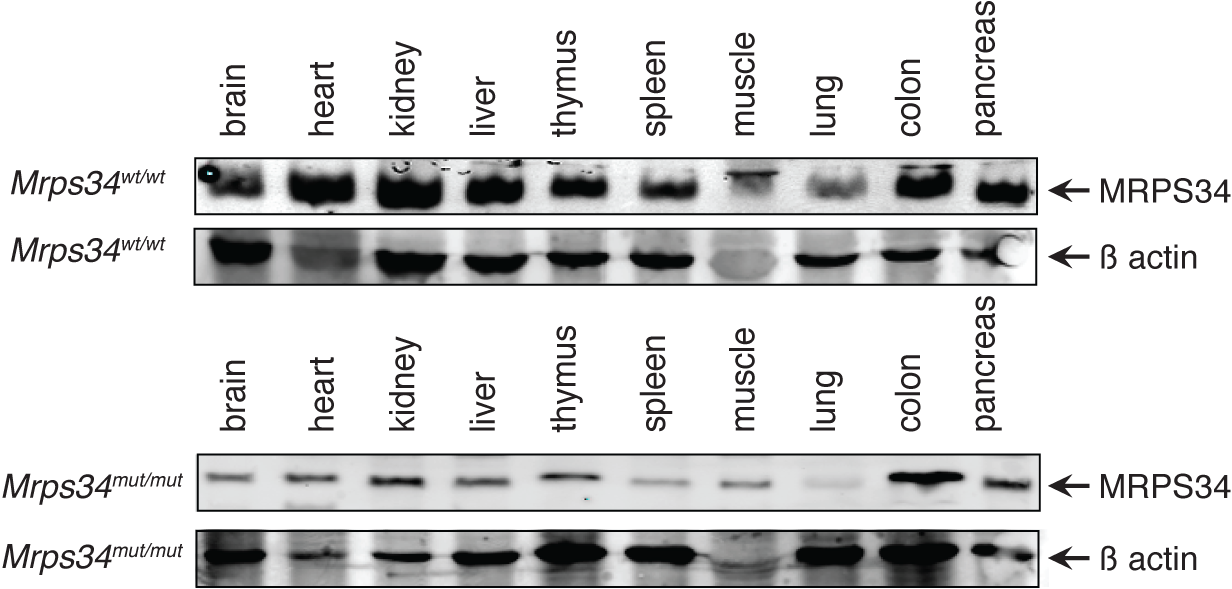

Supplement: S1 Fig — The distribution of MRPS34 and β-actin in tissue homogenates from Mrps34 wt/wt and Mrps34 mut/mut mice was determined by immunoblotting. (TIF) [file pgen.1005089.s002.tif]

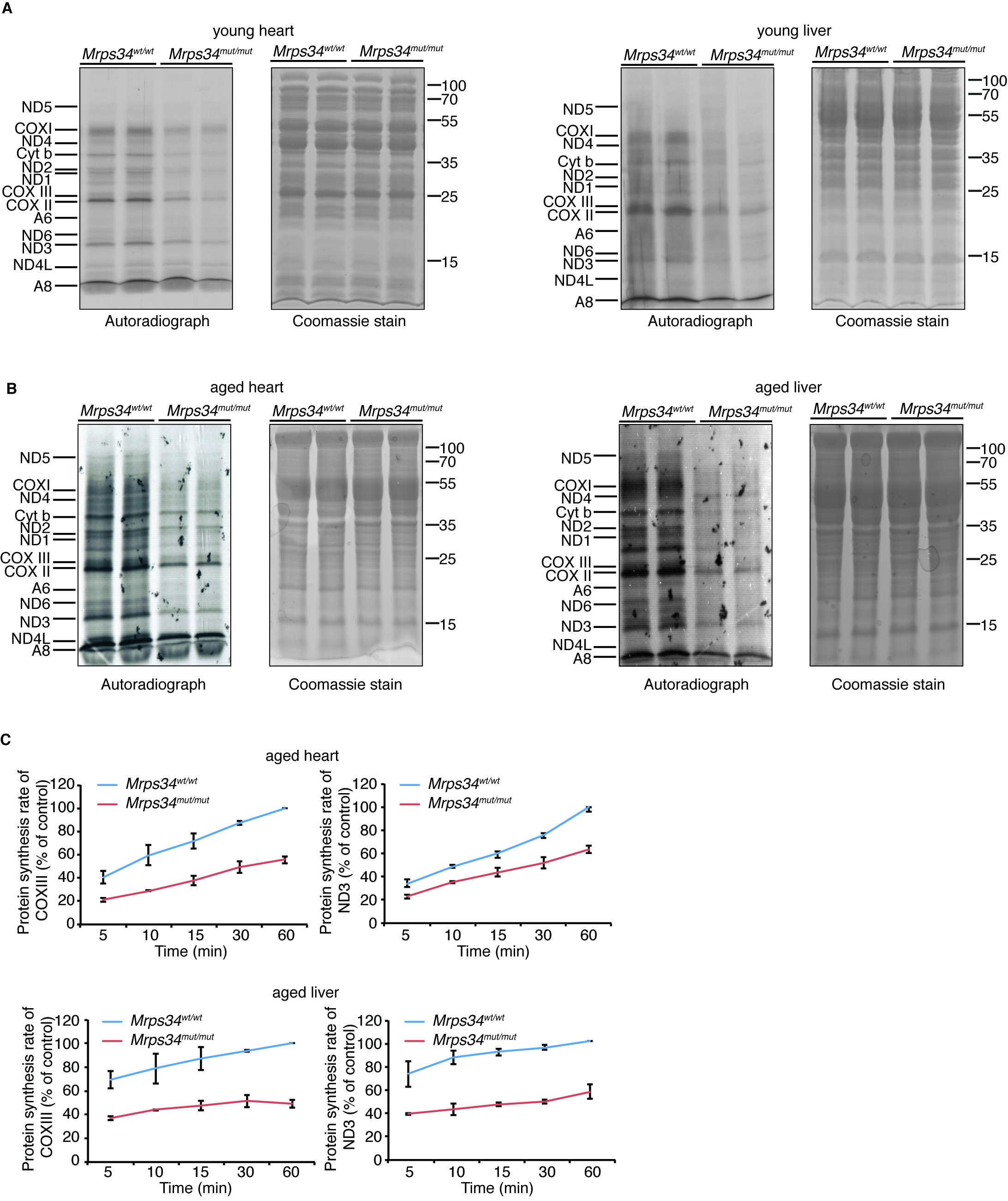

Supplement: S2 Fig — Protein synthesis of mitochondrially encoded proteins was measured in heart and liver mitochondria from young (A) and old (B) Mrps34 wt/wt (n = 5) and Mrps34 mut/mut (n = 5) by pulse incorporation of 35S-labeled methionine and cysteine after 1 hour. Equal amount of cell lysate protein (50 μg) was separated on SDS polyacrylamide gels and stained with Coomassie blue. The data shown are representative results of at least five different experiments. (C) Quantification of the rate of translation measured by pulse incorporation of 35S-labelled methionine and cysteine over time of COXIII and ND3 in heart and liver mitochondria from aged mice. Data are means ± SEM of three separate experiments. (TIF) [file pgen.1005089.s003.tif]

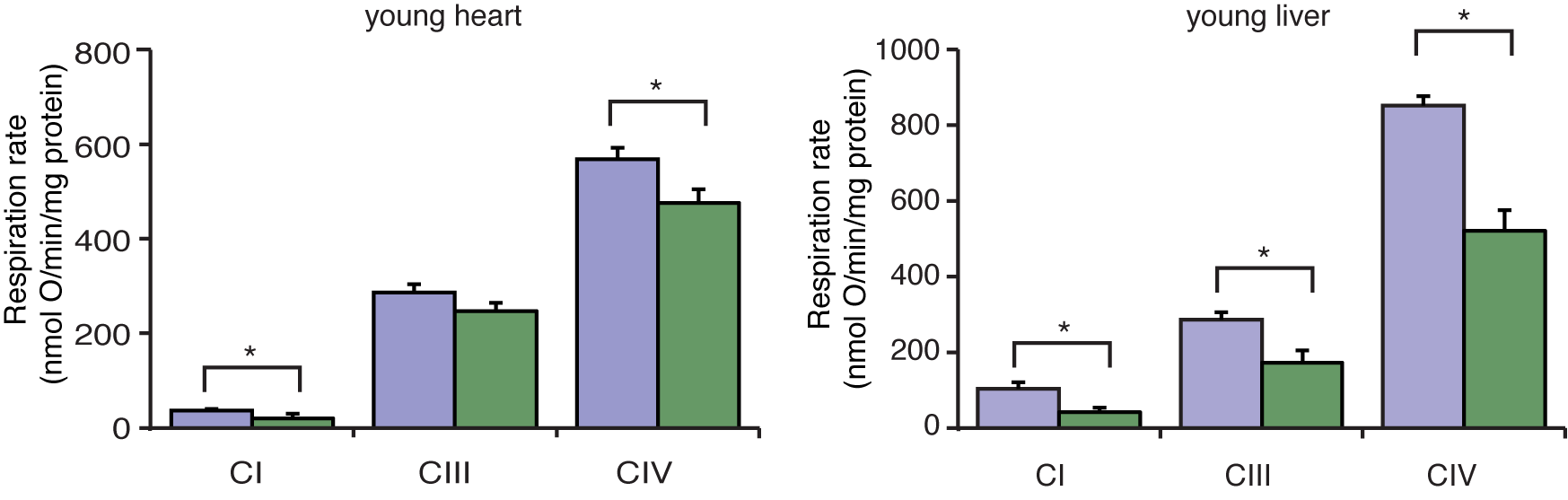

Supplement: S3 Fig — State 3 and 4 respiration was measured in mitochondria isolated from hearts and livers of young Mrps34 wt/wt and Mrps34 mut/mut mice using an OROBOROS oxygen electrode. Data are means ± SEM of three separate experiments; *, p < 0.05 compared with control treatments by a 2-tailed paired Student’s t test. (TIF) [file pgen.1005089.s004.tif]

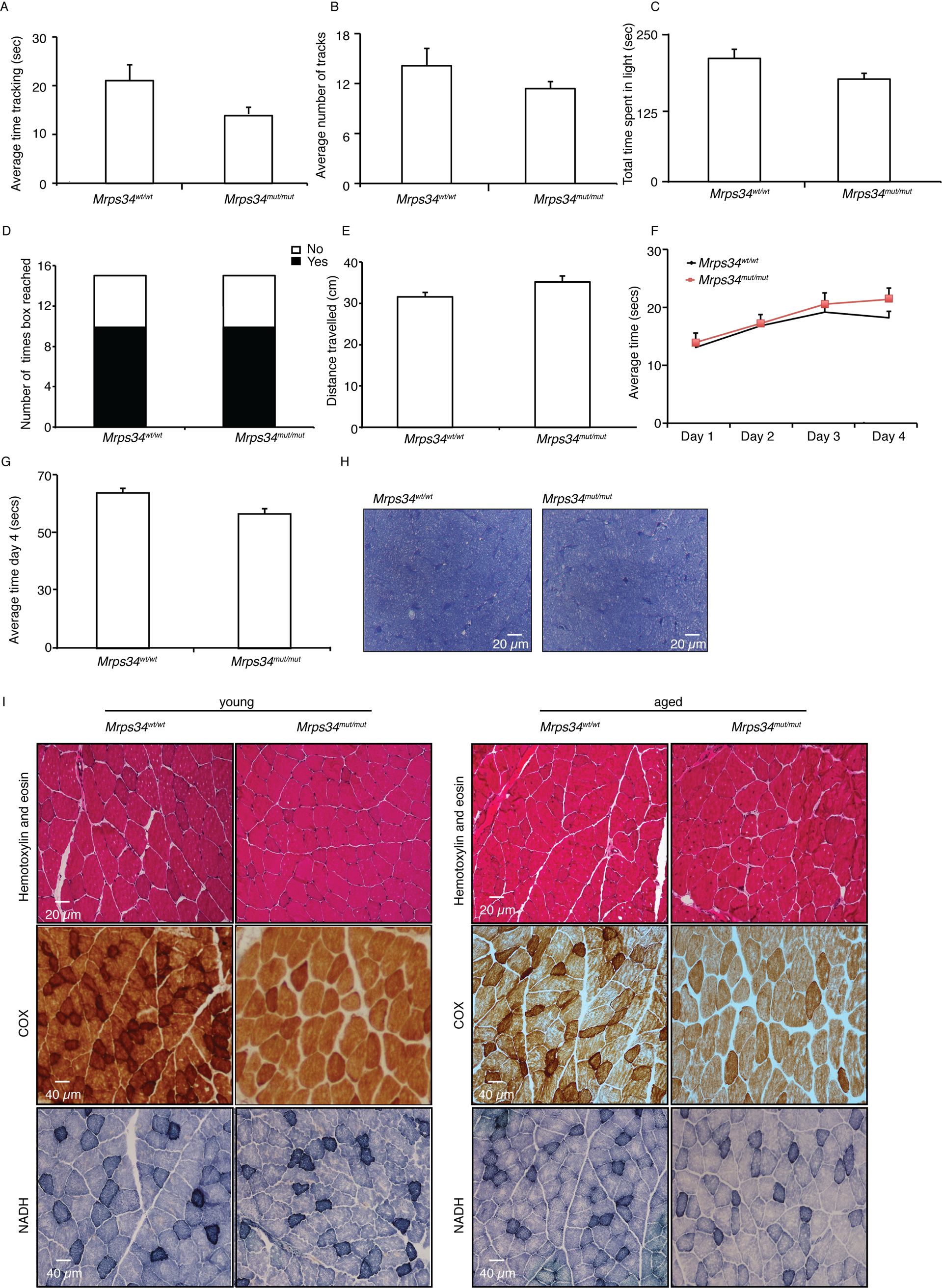

Supplement: S4 Fig — (A) Comparison of Mrps34 wt/wt (n = 5) and Mrps34 mut/mut (n = 5) tracking ability using optokinetic drum, measured in number of time spent tracking in seconds. (B) Comparison of Mrps34 wt/wt and Mrps34 mut/mut tracking ability measured in number of tracks performed. (C) Comparison of time spent in light versus dark in Mrps34 wt/wt (n = 5) and Mrps34 mut/mut (n = 5) mice measured in seconds. (D) Quantitation of behavioral studies evaluating number of times the box was reached in hanging wire experiments comparing Mrps34 wt/wt (n = 5) and Mrps34 mut/mut (n = 5) mice. (E) Quantitation of behavioral studies evaluating distance travelled along the wire in hanging wire experiments comparing Mrps34 wt/wt and Mrps34 mut/mut mice. (F) Rotarod results measured in seconds spent on the rotorrod over 4 days to show improvement and learning ability. (G) Time spent on the rotarod over 4 days to analyze motor function and learning ability. (H) Cresyl violet/toluidine blue staining of optic nerves from Mrps34 wt/wt (n = 5) and Mrps34 mut/mut (n = 5) mice visualized at 100x magnification. (I) Muscle sections cut at 10 μm thickness were stained with Haematoxylin and Eosin, COX and NADH from young and aged Mrps34 wt/wt (n = 9) and Mrps34 mut/mut (n = 9) mice and visualized at 40X magnification. (TIF) [file pgen.1005089.s005.tif]
